# A multispecies approach to manage effects of land cover and weather on upland game birds

**DOI:** 10.1002/ece3.7034

**Published:** 2020-11-19

**Authors:** Alexander R. Schindler, David A. Haukos, Christian A. Hagen, Beth E. Ross

**Affiliations:** ^1^ Department of Forestry and Environmental Conservation Clemson University Clemson SC USA; ^2^ U.S. Geological Survey, Kansas Cooperative Fish and Wildlife Research Unit Kansas State University Manhattan KS USA; ^3^ Department of Fisheries and Wildlife Oregon State University Corvallis OR USA; ^4^ U.S. Geological Survey, South Carolina Cooperative Fish and Wildlife Research Unit Clemson University Clemson SC USA

**Keywords:** conservation, land cover change, threshold models, upland game birds, weather change

## Abstract

Loss and degradation of grasslands in the Great Plains region have resulted in major declines in abundance of grassland bird species. To ensure future viability of grassland bird populations, it is crucial to evaluate specific effects of environmental factors among species to determine drivers of population decline and develop effective conservation strategies. We used threshold models to quantify the effects of land cover and weather changes in "lesser prairie‐chicken" and "greater prairie‐chicken" (*Tympanuchus pallidicinctus* and *T. cupido*, respectively), northern bobwhites (*Colinus virginianus*), and ring‐necked pheasants (*Phasianus colchicus*). We demonstrated a novel approach for estimating landscape conditions needed to optimize abundance across multiple species at a variety of spatial scales. Abundance of all four species was highest following wet summers and dry winters. Prairie chicken and ring‐necked pheasant abundance was highest following cool winters, while northern bobwhite abundance was highest following warm winters. Greater prairie chicken and northern bobwhite abundance was also highest following cooler summers. Optimal abundance of each species occurred in landscapes that represented a grassland and cropland mosaic, though prairie chicken abundance was optimized in landscapes with more grassland and less edge habitat than northern bobwhites and ring‐necked pheasants. Because these effects differed among species, managing for an optimal landscape for multiple species may not be the optimal scenario for any one species.

## Graphical abstract

We quantified the effects of land cover and weather changes in Galliformes in Kansas. We found evidence of nonlinear, threshold effects of land cover change in abundance of all four focal species, suggesting that the presence of both grassland and cropland is important in the landscape to simultaneously maintain these populations.

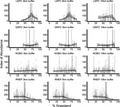

## 1 INTRODUCTION

The Great Plains of the United States is an important grassland system supporting many plant and animal species but is one of the most endangered grassland systems on Earth (Samson & Knopf, 1994; Samson et al., 2004). Grasslands in the Great Plains, consisting of short, mixed, and tallgrass species along a west to east precipitation gradient, have been steadily declining in quantity and quality since widespread European settlement of the area beginning with the Homestead Act in 1862 (Cully et al., 2003; Engle et al., 2008; Samson & Knopf, 1994). By the early 2000s, an estimated 70% of Great Plains grassland had been lost (Samson et al., 2004). Such landscape losses have caused dramatic declines in grassland bird populations, including many endemic species (Coppedge et al., 2001; Rosenberg et al., 2019; Sauer et al., 2013).

Grassland birds in the United States are declining faster than any other avian guild (BirdLife International, 2018; Brennan & Kuvlesky, 2005; Rosenberg et al., 2019). For many native grassland specialist species, these losses are a result of anthropogenic‐driven habitat loss. As agriculture became more prevalent and intensified in the Great Plains, vast areas of grasslands were converted to croplands and much of the remaining grasslands were intensively grazed (Augustine et al., 2019). This conversion of land cover paired with practices such as pesticide use, intensive unmanaged grazing, invasive plants, declining nutritional quality, and inappropriate burning tactics led to changes in the landscape that negatively affected both habitat quantity and quality for grassland birds (Samson et al., 2004). Energy development, including oil, natural gas, and wind energy, has also increased in the Great Plains and further contributed to loss and degradation of grassland habitat (Bartuszevige & Daniels, 2016). Government‐sponsored programs have helped slow and reverse loss of native grassland throughout the Great Plains (Spencer et al., 2017). The most prevalent of these is the Conservation Reserve Program (CRP), a cost‐share program under the United States Department of Agriculture Farm Services Agency in which landowners agree to establish perennial grass cover on former row‐crop fields for contract periods of 10–15 years in exchange for rental payments (Farm Service Agency, 2019; Ribic et al., 2009; Spencer et al., 2017).

In addition to landscape characteristics, climatic drivers also affect avian populations in the Great Plains (Peterson, 2003). Climate change can directly affect avian populations through physiological limitations leading to changing survival and recruitment rates (Carroll et al., 2015, 2016; Grisham et al., 2016; Laskowski et al., 2017; Raynor et al., 2019; Root, 1988). Climate change can additionally affect avian populations through modifying potential habitat, leading to shifts in species’ ranges, reductions in population abundance, and, eventually, local extinctions (Root, Price, et al., 2003; Thomas et al., 2006; Virkkala et al., 2008). However, conservation strategies often do not take projected climate change into consideration (Langham et al., 2015). More information is needed to understand specific species’ responses to differing weather conditions to project species’ response to potential changes in climate.

Conservation strategies that focus on benefits to multiple species simultaneously are often most effective, especially in areas with numerous species of conservation concern (Early & Thomas, 2007; Root, Akçakaya, et al., 2003; Zipkin et al., 2010). The concepts of “umbrella species” or “indicator species” are often used in multispecies conservation planning. These terms refer to species that have habitat requirements similar to those of many other species but have more extensive spatial needs (Suter et al., 2002). Developing management strategies to conserve habitat of an umbrella or indicator species would therefore indirectly benefit many other species as well. While this approach is useful in some regions under some specific conservation goals, habitat and resource needs of most species rarely perfectly overlap, resulting in many umbrella species plans providing suboptimal solutions for the species of interest (Carlisle et al., 2018; Crosby et al., 2015). In these cases, an alternative solution may be to manage for an optimal landscape that may not be the best scenario for any one species but beneficial to the greatest number of focal species (Holzkämper et al., 2006; van Teeffelen et al., 2008). This approach may prove useful in the Great Plains, where many species of conservation concern have different, and oftentimes conflicting, resource and habitat needs.

Lesser and greater prairie chickens (*Tympanuchus pallidicinctus* and *T. cupido*, respectively), northern bobwhites (*Colinus virginianus*), and ring‐necked pheasants (*Phasianus colchicus*) are all economically important upland game birds in the Great Plains, but have experienced recent declines in portions of the area (Hernández et al., 2013; Ross et al., 2016a, 2016b; Sauer et al., 2013). Lesser and greater prairie chickens have experienced large declines throughout their respective ranges, and the management goals for these species are focused on reversing declines to ensure long‐term persistence of the species (Hagen et al., 2004; McNew et al., 2011; Van Pelt et al., 2013). Northern bobwhites are also a species of conservation concern throughout much of their range, although northern bobwhites in the Great Plains have not experienced the same severity of decline as populations in the eastern United States (Brennan, 1991; Hernández et al., 2013; Sauer et al., 2013). Management goals for northern bobwhites involve increasing range‐wide population densities, with particular focus on restoration to levels that can sustain harvest (The National Bobwhite Technical Committee, 2011). Ring‐necked pheasants, while not a native species to the United States, are intensively managed with the goal of sustaining populations to support continued harvest (Midwest Pheasant Study Group of the Midwest Association of Fish & Wildlife Agencies 2013). Using an optimal landscape approach may help managers construct habitat conditions that maximize abundance of all four upland game birds simultaneously. Managers could similarly develop plans for combinations of species (e.g., for all native species of conservation concern) depending on conservation objectives.

Lesser and greater prairie chickens are obligate grassland birds that require landscapes with large patches of mid‐ and tall grasses (Haukos & Zavaleta, 2016; Jones, 1963; McNew, Gregory, et al., 2012; McNew, Prebyl, et al., 2012). Northern bobwhites are mainly found in landscapes containing a variety of early successional habitats, including perennial grasses, forbs, shrubs, and agricultural fields (Brennan, 1991; Roseberry & Sudkamp, 1998). Ring‐necked pheasants heavily rely upon agricultural lands in addition to grasslands (Gabbert et al., 1999; Hagen et al., 2007). Changes in habitat quantity and quality due to conversion of grassland to cropland, degradation of grassland through grazing, increasing energy infrastructure, and fluctuations in CRP enrollment are all major drivers of population change in these species, but differences in life history likely result in the severity of these effects varying across species and spatial scale (Brennan, 1991; Fuhlendorf et al., 2002; Haukos & Zavaleta, 2016; McNew, Gregory, et al., 2012; McNew, Prebyl, et al., 2012; Sauer et al., 2013). Severe weather conditions also negatively affect populations of these birds, with extreme summer temperatures and drought leading to decreased nest success (Carroll et al., 2015, 2017; Grisham et al., 2016; Laskowski et al., 2017; Ross et al., 2016b) and extreme winter temperatures and precipitation leading to decreased survival (Janke et al., 2017; Perkins et al., 1997; Peterson, 2016).

It is likely these species exhibit nonlinear responses to habitat changes, and lesser prairie chickens exhibit a “threshold” response to a gradient of cropland to grassland on the landscape (Ross et al., 2016a). The estimation of threshold responses for other similar species would allow managers to quantify change points at which populations will likely decrease or increase in response to habitat change. Moreover, traditional approaches to quantifying change points (e.g., generalized additive models or quadratic effects incorporated into linear models) involve detection of change points through visual estimation rather than explicit quantification with associated uncertainty (Powell et al., 2017), which can have limited practical applications (Toms & Villard, 2015). Implementing models with change points in a Bayesian hierarchical framework allows the estimation of change points and the ability to incorporate observation error (Wagner & Midway, 2014). The ability to estimate change points would also aid managers in identifying landscape characteristics that optimize abundance of multiple species. When habitat needs of multiple species do not perfectly overlap (i.e., change points differ among species), managers could estimate a range of habitat characteristics (i.e., values between differing change points) that benefit the greatest number of focal species.

We examined the effects of land cover and weather on populations of upland game birds in Kansas. We quantified the effects of percent grassland, edge density of grassland patches, summer temperature and drought, and winter temperature and precipitation on abundance of lesser and greater prairie chicken, northern bobwhite, and ring‐necked pheasant populations using hierarchical models in a Bayesian framework across a gradient of fine to broad spatial scales. These analyses will provide valuable context to managers and aid in optimizing conservation and management efforts for multiple species.

## 2 METHODS

### 2.1 Study area

**FIGURE 1:**
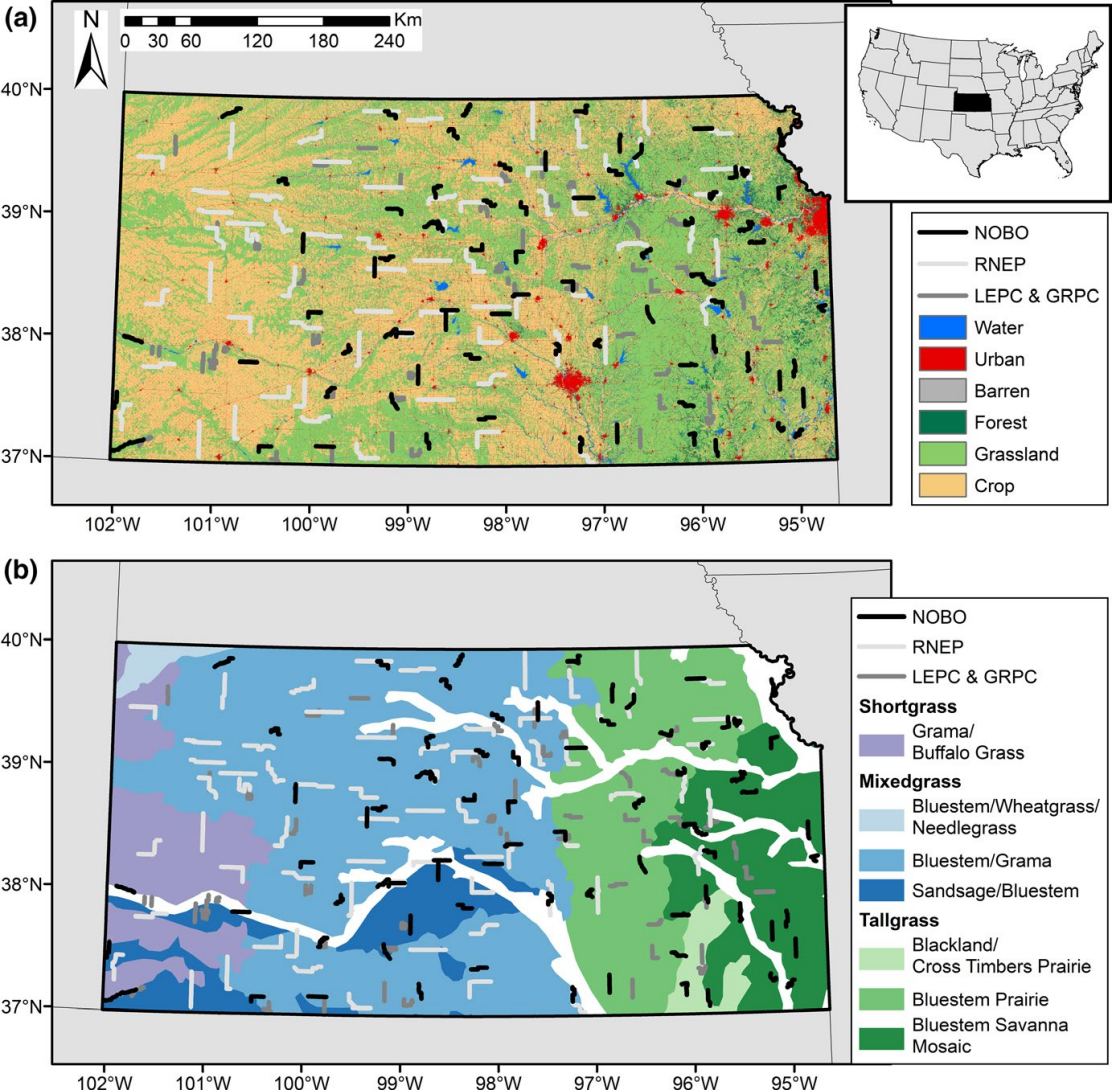
Distribution of route locations from annual count surveys conducted by the Kansas Department of Wildlife, Parks, and Tourism in Kansas for lesser and greater prairie chickens (LEPC &amp; GRPC), northern bobwhites (NOBO), and ring‐necked pheasants (RNEP) conducted across (a) land cover (created with data from U.S. Geological Survey, 2014) and (b) potential natural vegetation (created with data from Kuchler, 1964, grassland classes summarized based on Augustine et al., 2019)

We analyzed lesser and greater prairie chicken, northern bobwhite, and ring‐necked pheasant count data across Kansas. Vegetation in the study area largely consisted of grassland (both grazed and ungrazed) and cropland land cover types. Grasslands included both native grasslands and cropland removed from production and converted back to grassland under the CRP (Spencer et al., 2017). Native grasses included short (e.g., *Bouteloua dactyloides and B. gracilis*), mixed (e.g., *B. dactyloides*, *B. gracilis*, *B. curtipendula*, *Andropogon gerardii*, *Panicum virgatum*, *Schizachyrium scoparium*, *Sporobolus compositus*, and *Sorghastrum nutans*), and tall grasses (e.g., *A. gerardii, P. virgatum, S. nutans*) along a west‐to‐east precipitation gradient (Figure 1b; Augustine et al., 2019; Kuchler, 1964). CRP lands contained a variety of native, mixed‐grass species, as well as Old World Bluestems (*Bothriochloa* spp.) (Van Pelt et al., 2013). Agriculture primarily included corn (*Zea mays*), cotton (*Gossypium* spp.), sorghum (*Sorghum* spp.), soybeans (*Glycine* max), wheat (*Triticum aestivum*), and alfalfa (*Medicago sativa*), as well as pasture for cattle production (United States Department of Agriculture et al., 2018). Study sites were Kansas Department of Wildlife, Parks, and Tourism survey routes for each focal species. These survey routes occurred across Kansas, representing the majority of land use and vegetation types found in the state (Figure 1a; see Pitman, 2014; Prendergast, 2018a, 2018b for additional route information).

### 2.2 Count surveys

The Kansas Department of Wildlife, Parks, and Tourism conducted count surveys on a collection of routes for all four focal species each year during the breeding season using roadside surveys (Table 1). Biologists surveyed each lesser and greater prairie chicken route twice each season and surveyed each northern bobwhite and ring‐necked pheasant route once a season. Each survey route consisted of 11 stops. On northern bobwhite and ring‐necked pheasant routes, biologists conducted auditory surveys at each stop, with observers counting the number of calling males or number of crowing calls made by males, respectively (Prendergast, 2018a, 2018b). On prairie chicken routes, observers conducted auditory surveys at each route to identify prairie chicken lek (a breeding ground, defined as group of 3 or more chickens) locations. Observers flushed each lek and visually counted all prairie chickens at the lek immediately following the auditory surveys (Pitman, 2014). Time of day, survey period, listening duration, and route length varied between species (Table 1). Consistent prairie chicken survey routes were established in 1978. We only used greater prairie chicken count data beginning in 1996 due to a lack of land cover data in the greater prairie chicken range in prior years. Northern bobwhite survey routes were established in 1998. Ring‐necked pheasant survey routes were established in 1997. To better assess the effects of land cover and weather on avian populations on the appropriate scales, we summed count data of all 11 stops on each route for each visit, in the case of prairie chicken surveys, and of all 11 stops on each route for each year, in the case of northern bobwhite and ring‐necked pheasant surveys.

**TABLE 1:** Descriptions of annual upland game bird count surveys conducted by the Kansas Department of Wildlife, Parks, and Tourism for lesser and greater prairie chickens (LEPC and GRPC), northern bobwhites (NOBO), and ring‐necked pheasants (RNEP). Observers conducted surveys along routes consisting of 11 stops at 1‐ to 2‐mile (1.6–3.2 km) intervals

Species	Survey years	Dates of survey	Time of surveys	Listening duration (min)	Route length (km)
LEPC	1978–2014	20 March–20 April	30 min before sunrise–90 min after sunrise	3	16
GRPC	1996–2014	20 March–20 April	30 min before sunrise–90 min after sunrise	3	16
NOBO	1998–2015	1 June–16 June	Sunrise–completion of route	5	16
RNEP	1997–2015	25 April–15 May	45 min before sunset–completion of route	2	32

Note

Routes were located across each of the species’ respective ranges in Kansas. Shown are the species counted in each set of surveys, years of survey data used in analyses, range of dates over which surveys were conducted each year, range of times over which surveys were conducted each day over the annual survey period, duration of each count at each stop along a route, and each route length.

### 2.3 Environmental variables

To assess the effects of land cover change in abundance of these four focal species, we acquired land cover data from several sources. For land cover in the lesser prairie chicken range, we used LANDSAT imagery for 1978, 1985, 1988, 1994, 2003, and 2013. Using techniques described in Spencer et al. (2017), we classified land cover as grassland, cropland, urban, or water at a 30‐m resolution. For land cover in the ranges of the other three focal species, we obtained data for the entire state of Kansas from the National Land Cover Database for 2001, 2006, and 2011 (U.S. Geological Survey, 2014). We similarly classified land cover in this data set as grassland, cropland, urban, or water at a 30‐m resolution. We were unable to separate native grassland and land enrolled in the CRP in remote sensing data, so our grassland classification referred to a combination of these areas. Enrollment in CRP occurred in 1986 and 1987 with re‐enrollment in 1996 and 1997 and again in 2006 or 2011. Changes in land enrolled in CRP are the major drivers of land cover change in upland game bird habitat in Kansas (Spencer et al., 2017). We therefore assumed land cover in the buffered areas remained constant between CRP contract years to fill in gaps in land cover data.

We estimated the effects of land cover by calculating the percentage of land covered by grassland and the edge density of grassland patches in varying buffer sizes around each survey route for each year of land cover data. Home ranges varied among focal species, so we used buffer sizes of 3, 5, and 10 km around the survey routes to assess the effects of land cover on populations at a variety of spatial scales (Applegate et al., 2002; Haukos & Zavaleta, 2016; Janke & Gates, 2012; Patten et al., 2011). We used Fragstats version 4 (McGarigal et al., 2012) to calculate the percentage of land covered by grassland in the buffered area around each route in each year. We also used Fragstats to calculate edge density (ED, measured in m/ha) of grassland patches by summing the lengths of all edge segments of grassland and dividing by the total grassland area in each buffered landscape for each year. We paired count data with land cover data from the associated CRP contract period (e.g., percent grassland and ED calculated from NLCD 2001 data affected abundance of greater prairie chickens, northern bobwhites, and ring‐necked pheasants during each year of the 1996–2005 contract period).

To assess the effects of weather on abundance of the four focal species, we obtained historical weather data from the National Climatic Data Center for each of the 9 climate regions in the state of Kansas (Vose et al., 2014). Weather data contained information on summer temperatures and drought, and winter temperatures and precipitation. We used the Palmer Drought Severity Index (PDSI) to quantify summer drought. We created a drought covariate by averaging the PDSI values for June, July, and August each year, for each climate region, and implemented a 1‐year lag effect (Ross et al., 2016a, 2016b). We created a covariate for summer temperature by selecting the highest monthly maximum temperature (TMAX) from values in June, July, and August each year, for each climate region, and applied a similar 1‐year lag effect. We therefore expected positive summer PDSI and cooler summer TMAX values in year *t* – 1 would improve reproductive success, reflected in higher abundance in year *t* due to a larger number of first‐year breeders. We created a covariate for winter temperature by selecting the lowest monthly minimum temperature (TMIN) from values during December, January, and February preceding a breeding season. We also used the precipitation index (total precipitation for a month; PCP) to quantify winter precipitation. We created a covariate for winter precipitation by averaging the PCP values for December, January, and February preceding each breeding season for each climate region (i.e., averaging the three monthly precipitation totals). We therefore expected lower PCP and higher TMIN values in December of year *t* ‐ 1 and January and February of year *t* would improve survival, reflected in higher abundance in year *t*.

### 2.4 Statistical modeling

Addressing within‐year variation in counts of lekking individuals due to imperfect detection is important in preventing biases in annual abundance estimates (Sadoti et al., 2016). While many studies have identified the need to address imperfect detection of lekking species to improve abundance estimates, these studies often rely on maximum lek counts to adjust abundance estimates (Garton et al., 2011; Hancock et al., 1999). More recently, several studies have incorporated methods that use repeated counts within a survey season to directly estimate detection probability (McNew, Prebyl, et al., 2012; Ross et al., 2016a, 2016b). We implemented hierarchical models in a Bayesian framework (Royle, 2004) to estimate and quantify the effects of land cover and weather on lesser and greater prairie chicken abundance across the respective ranges of these species in Kansas. These models allowed us to account for imperfect detection of individuals by using repeated counts within a survey season and required 4 assumptions that were met in this study: (1) The population of interest was closed during a survey season, (2) detection probability was constant for all individuals within a survey season, (3) abundance and detection probability were adequately described by the chosen parametric distribution, and (4) there were no false positives such as double counts (Kéry & Schaub, 2012). While variation in lek attendance within a breeding season may have affected the population closure assumption in our study, lesser and greater prairie chicken movements are limited in spring and intervals among repeated counts within a season were short, so these effects were likely small (Haukos & Zavaleta, 2016; McNew, Prebyl, et al., 2012; Nooker & Sandercock, 2008).

Data in our models were specified as coming from a binomial distribution

(1) $$y_{i,j,t}∼\text{Bin}\left(N_{i,t},p_{i,j,t}\right)$$

where the *y_i,j,t_*, consisting of count data from lek surveys at route *i*, visit *j*, and year *t*, is distributed binomially with parameters *N_i,t_*, the estimated abundance on leks, and *p_i,j,t_*, the probability of detection. Due to count survey methodology, we were not able to separate detection probability associated with identifying leks from detection probability associated with detecting individuals on a lek. The probability of detection in all prairie chicken models therefore refers to this combined detection (Ross et al., 2016a, 2016b). Additionally, while detection probability varied by site, time, and occasion, we were unable to incorporate information on variation in detection probability related to covariates as these data were missing from portions of the data sets.

We constructed process models to describe change in male prairie chicken abundance on leks, which we assumed to be representative of changes in prairie chicken populations as a whole. We modeled the abundance on leks as coming from a negative binomial distribution to account for overdispersion in the count data.

(2) $$N_{i,t}∼\text{NegBin}\left(s_{i,t},r\right)$$

where *r* was the overdispersion parameter, *s_i,t_* the probability of success, and mean abundance *μ_i,t_* = *r*(1 – *s_i,t_*)/*s_i,t_*. This parameterization allowed for the variance in abundance to be larger than the mean (*μ_i,t_*). We implemented two piecewise linear models (Qian, 2014; Qian & Cuffney,  that allowed for thresholds or change points along the gradient of percent grassland and edge density with linear effects for all weather covariates. These piecewise linear models, or “threshold models,” were defined as

(3) $$z_{i,t}=\log\left(\mu_{i,t}\right)=\beta_{0}+\beta X+\left(\beta_{5}+\delta I\left(x_{\text{GRASS}}‐\phi \right)\right)\left(x_{\text{GRASS}}‐\phi \right)+\varepsilon_{i,t}$$

and

(4) $$z_{i,t}=\log\left(\mu_{i,t}\right)=\beta_{0}+\beta X+\left(\beta_{5}+\delta I\left(x_{\text{ED}}‐\phi \right)\right)\left(x_{\text{ED}}‐\phi \right)+\varepsilon_{i,t}$$

where *β*_0_ was an intercept, ***β*** a vector for the coefficients *β*_1_–*β*_4_ for PDSI, PCP, TMAX, and TMIN, and *β*_5_ the coefficient for percent grassland (designated as GRASS) or the coefficient for ED. Our threshold model estimated two slopes: *β*_5_ which described the relationship of the variables before the threshold (*ϕ*) and an intensification coefficient, *δ*, that described the change in slope after the threshold. The indicator function, *I*(*a*) = 0 when *a* < 0 (*x*–*ϕ* < 0, that is, before reaching the threshold value) and *I*(*a*) = 1 when *a* ≥ 0 (*x*–*ϕ* ≥ 0, i.e., after reaching the threshold value). The *ε_i,t_* ~ *N*(0, *σ*) was random intercepts for route and time.

The northern bobwhite and ring‐necked pheasant surveys did not have repeated counts, and we could not estimate detection probability. Instead, in both these models, data were defined as

(5) $$y_{i,t}∼\text{NegBin}\left(s_{i,t},r\right)$$

where the *y_i,t_*, consisting of count data from auditory surveys at route *i* and year *t*, was distributed with a negative binomial distribution with *r* as the overdispersion parameter, *s_i,t_* the probability of success, and the mean abundance *μ_i,t_* = *r*(1–*s_i,t_*)/*s_i,t_*. We implemented the same piecewise linear models (Equations 3 and 4) for these species as well.

We used Markov chain Monte Carlo and a Gibbs sampler in JAGS 4.3.0 (Plummer, 2017) with the package runjags (Denwood, 2016) in program R version 3.4.3 (R Core Development Team, 2017) to obtain posterior distributions for all model parameters. We discarded at minimum the first 200,000 samples as burn‐in, used a thinning rate of 5, and saved at least 10,000 samples from 3 chains for all models. We evaluated convergence of chains with a Gelman–Rubin statistic (*R* < 1.1). We specified prior distributions as *β*_0_ ~ *N*(0, 10), ***β*** ~ *N*(0, 10), *β*_5_ ~ *N*(0, 10), *δ* ~ *N*(0, 10), *ε_i,t_* ~ *N*(0, 15), *r* ~ Gamma(1, 1), and *ϕ* ~ U(*l*, *u*), where l and u were the lower and upper values of the standardized percent grassland or edge density, respectively. We repeated all modeling for all four species using land cover data from the 3‐, 5‐, and 10‐km buffer sizes. To account for routes that were not surveyed in some years, we used estimated weather and land cover effects to predict abundance for routes each year with no count data to properly estimate changes in abundance among years. We assessed the fit of each model by comparing residuals and predicted values to a 1‐to‐1 line and calculated Bayesian *p*‐values by averaging the probability that the data were greater than the predicted values across the posterior distribution. We quantified the probabilities of significant covariate effects by calculating the percent of the posterior distributions for *β*_1‐5_ above (for positive effects) or below (for negative effects) 0. We similarly quantified the probabilities of percent grassland or edge density threshold effects (i.e., changes to *β*_5_ following the threshold point, *ϕ*) as the percent of the posterior distribution for *δ* above or below 0.

## 3 RESULTS

### 3.1 Population trends

**FIGURE 2:**
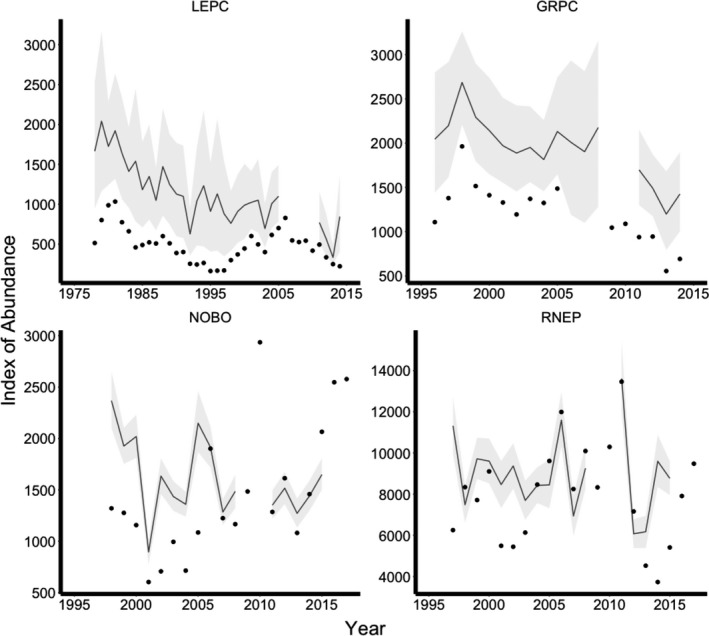
Estimates of total abundance from the models for lesser prairie chickens (LEPC), greater prairie chickens (GRPC), northern bobwhites (NOBO), and ring‐necked pheasants (RNEP) on surveyed routes in Kansas. Index of abundance represents the number of males on leks, calling males, and crowing calls summed across all routes for LEPC and GRPC, NOBO, and RNEP, respectively. Total counts from the surveys are shown as points and the 95% credible intervals of population estimates are shown in gray. Population estimates were not calculated in years where a lack of covariate data prevented abundance estimation

From 1978 to 2014, an average of 486.49 (*σ* = 215.76) lesser prairie chickens were observed per year among 17 routes. Estimated abundance indicated lesser prairie chickens on observed leks decreased by about 49.3% from 1978 to 2014, an average decrease of 1.4% per year (Figure 2). Detection probability for lesser prairie chickens had a mean of 0.68 (95% credible interval [CRI] = 0.56–0.78; Figure S1). From 1996 to 2014, an average of 1,209.50 (*σ* = 314.72) greater prairie chickens were observed per year among 33 routes. Estimated abundance indicated greater prairie chickens on observed leks decreased by about 30.3% between 1996 and 2014, an average decrease of 1.7% per year (Figure 2). Detection probability for greater prairie chickens had a mean of 0.67 (95% CRI = 0.63–0.72; Figure S2). From 1998 to 2015, an average of 1,459.70 (*σ* = 643.67) northern bobwhites were observed per year among 74 routes. Estimated abundance indicated calling northern bobwhite males on surveyed routes remained relatively constant from 1998 to 2015, although there was considerable interannual variation (Figure 2). From 1997 to 2015, an average of 7,959.81 (*σ* = 2,439.21) ring‐necked pheasant crowing calls were recorded among 66 routes. Estimated abundance indicated the number of ring‐necked crowing calls on surveyed routes remained relatively constant from 1997 to 2015, although there was also considerable interannual variation (Figure 2).

### 3.2 Threshold models

All threshold models for each species fit our data (Bayesian p‐values between 0.08 and 0.61). All species exhibited high probabilities of a significant linear response to at least one weather variable and high probabilities of a significant threshold response to both percent grassland and edge density, though specific effects varied by species and buffer size.

**FIGURE 3:**
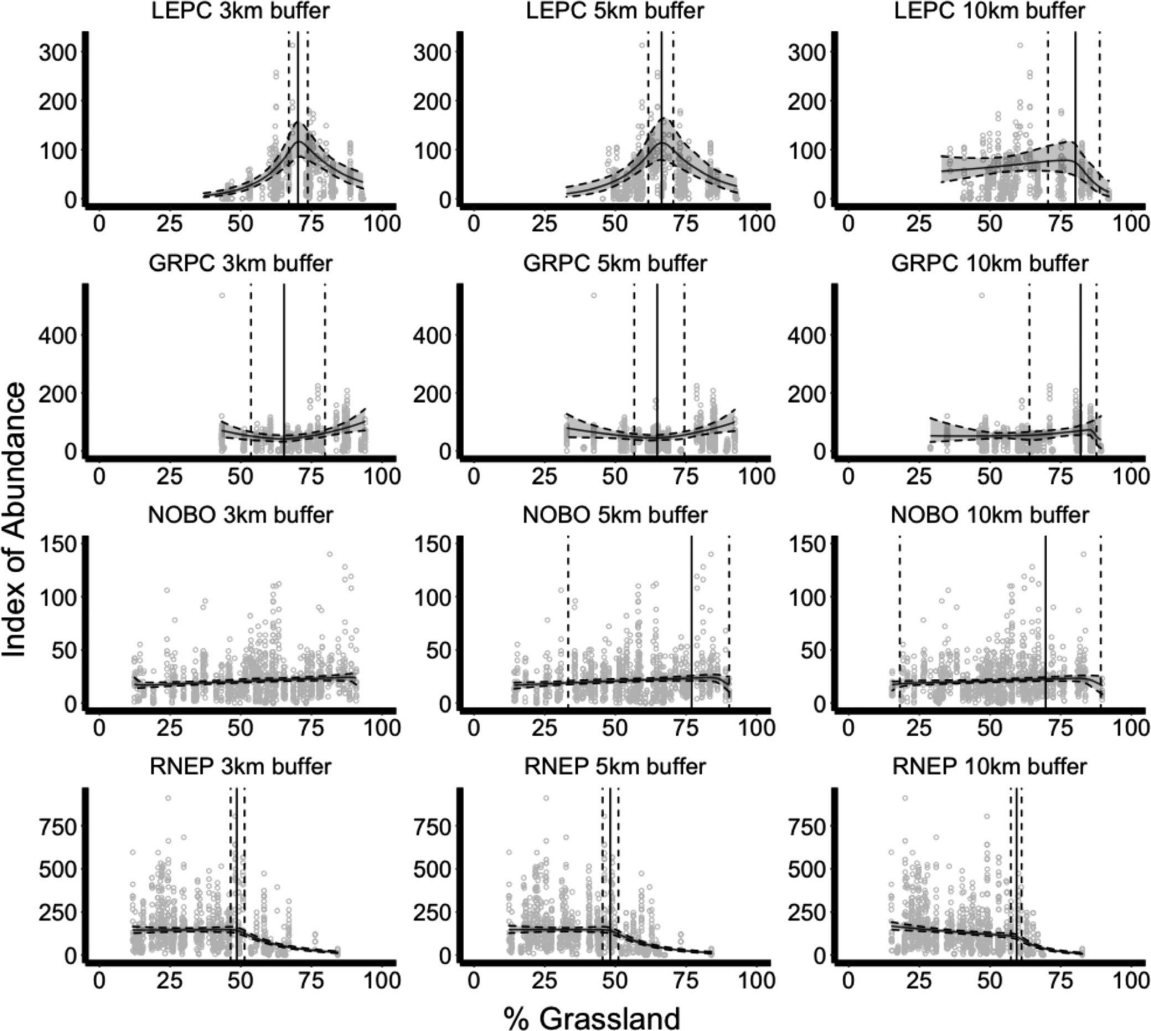
Changes in relative abundance of lesser prairie chickens (LEPC), greater prairie chickens (GRPC), northern bobwhites (NOBO), and ring‐necked pheasants (RNEP) in Kansas in response to percent grassland, with 95% credible intervals shown in gray between dashed lines. Index of abundance represents the number of males on leks, calling males, and crowing calls per route for LEPC and GRPC, NOBO, and RNEP, respectively. The threshold point is represented by a solid vertical line, and the 95% credible intervals of the threshold point are represented by vertical dashed lines. Results were constrained between the minimum and maximum percent grassland values observed

**FIGURE 4:**
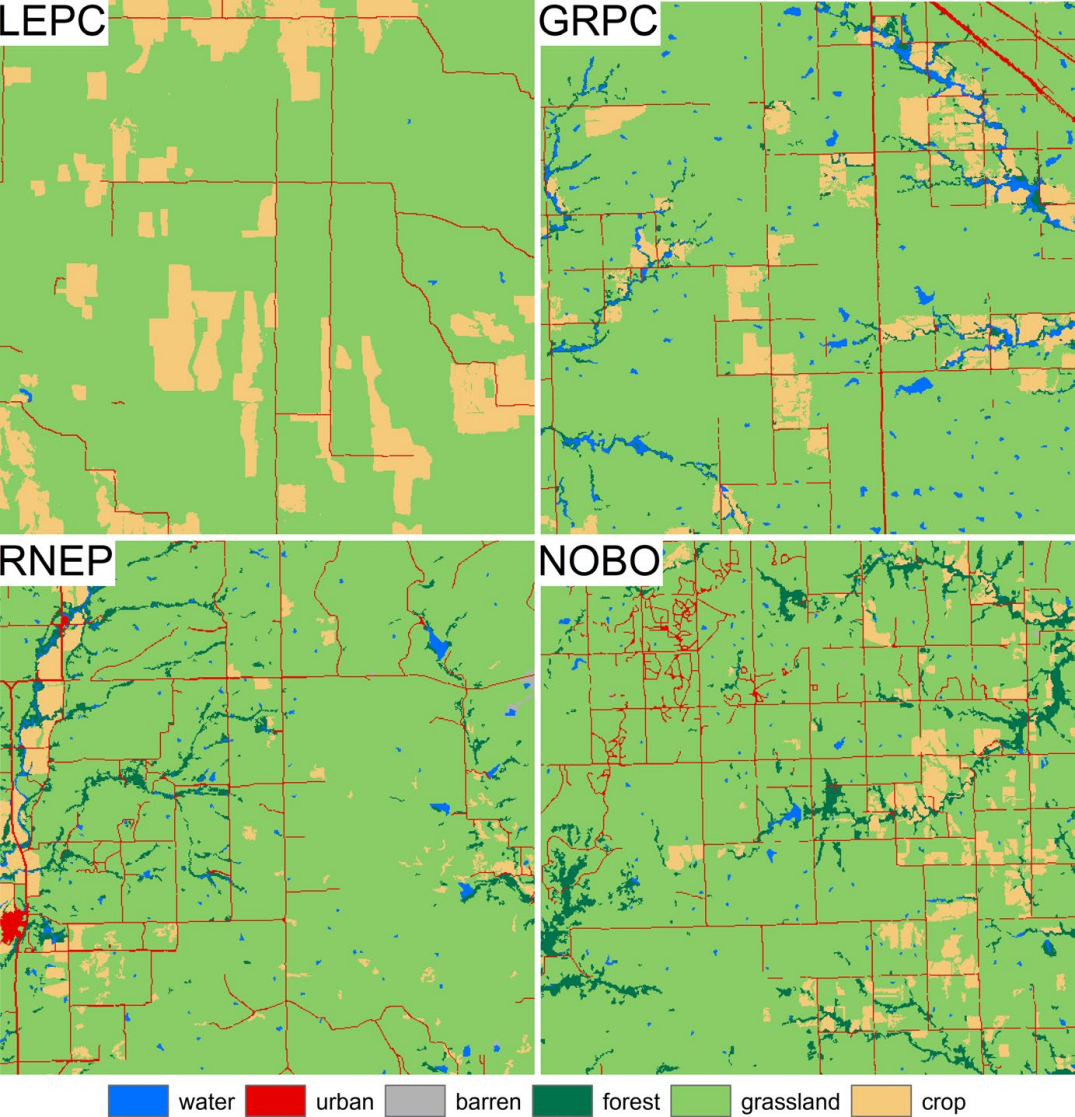
Examples of observed landscapes surrounding lesser prairie chicken (LEPC), greater prairie chicken (GRPC), northern bobwhite (NOBO), and ring‐necked pheasant (RNEP) survey routes that contained grassland configured for optimal abundance for each species of interest based on the results of edge density (ED) models. Examples containing similar percent grassland (79%–87%) were selected for visual comparison

**FIGURE 5:**
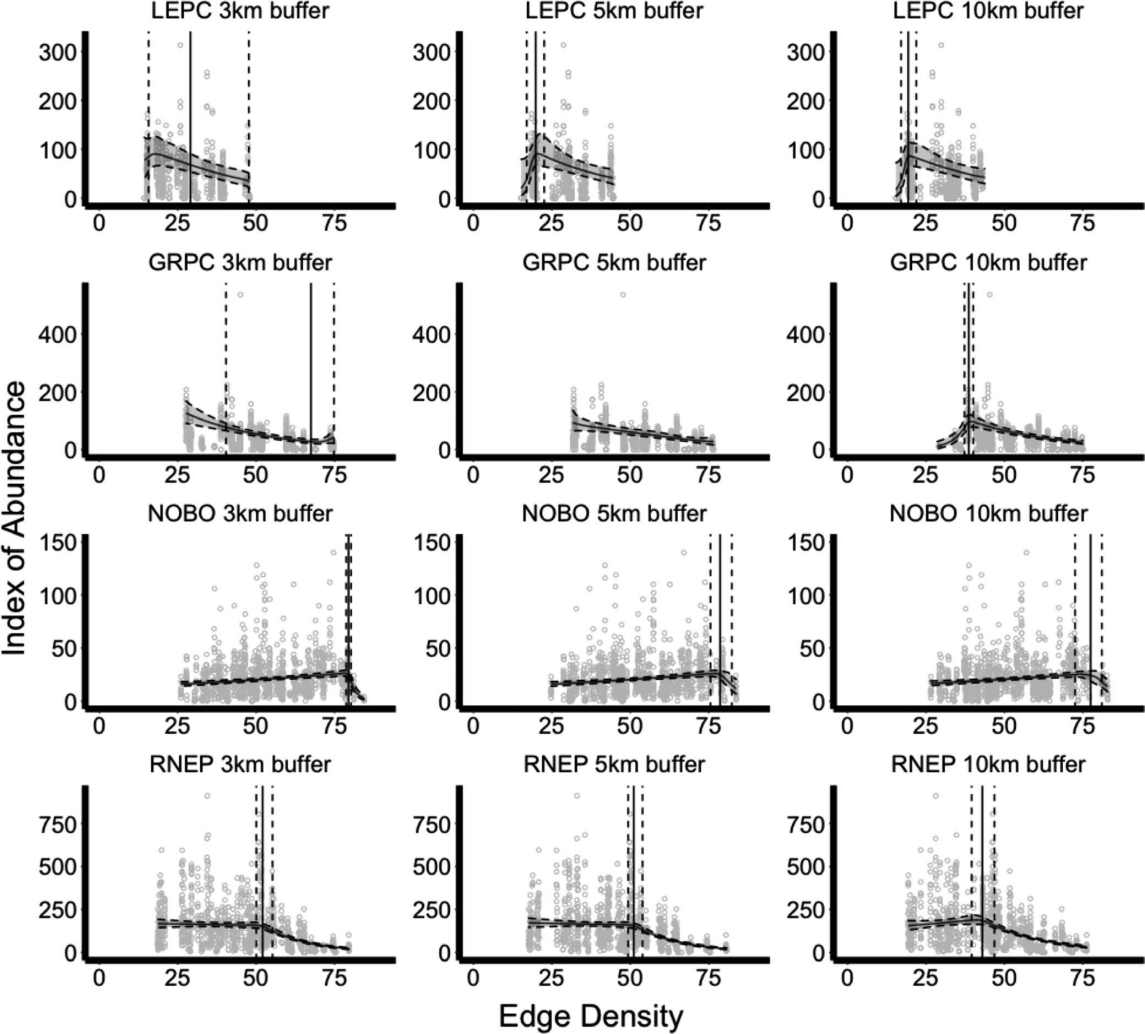
Changes in relative abundance of lesser prairie chickens (LEPC), greater prairie chickens (GRPC), northern bobwhites (NOBO), and ring‐necked pheasants (RNEP) in Kansas in response to edge density of grassland patches in m/ha, with 95% credible intervals shown in gray between dashed lines. Index of abundance represents the number of males on leks, calling males, and crowing calls per route for LEPC and GRPC, NOBO, and RNEP, respectively. The threshold point is represented by a solid vertical line, and the 95% credible intervals of the threshold point are represented by dashed lines vertical lines. Results were constrained between the minimum and maximum edge density values observed

Lesser prairie chicken abundance was highest in years following high summer PDSI (i.e., wetter summers; Pr(*β*_1_ > 0) = 0.851–0.961), low winter PCP (i.e., drier winters; Pr(*β*_2_ < 0) = 0.615–0.979), and low winter TMIN (i.e., cooler winters; Pr(*β*_4_ < 0) = 0.974 – 0.996) (Tables 2 and 3). Summer TMAX did not have a high probability of affecting lesser prairie chicken abundance (Pr(*β*_3_ < 0) = 0.427–0.627). At the 3‐, 5‐, and 10‐km spatial scales, lesser prairie chicken abundance initially increased with increasing grassland (Pr(*β*_5_ > 0) = 0.839–1) until specific threshold points (3 km: *ϕ* = 70.2%, 95% CRI = 67.0%–73.7%; 5 km: *ϕ* = 66.3%, 95% CRI = 61.7%–70.5%; 10 km: *ϕ* = 80.2%, 95% CRI = 70.5%–88.8% grassland), after which abundance decreased with increasing grassland (Pr(*δ* < 0) = 0.999–1) (Table 2, Figure 3). At the 3‐km spatial scale, lesser prairie chicken abundance did not initially differ with increasing edge density of grassland patches (Pr(*β*_5_ > 0) = 0.533) until the threshold point (*ϕ* = 29.0 m/ha, 95% CRI = 15.7–47.7 m/ha), after which abundance decreased with increasing edge density (Pr(*δ* < 0) = 0.911) (Table 3, Figure 5). At the 5‐ and 10‐km spatial scales, lesser prairie chicken abundance initially increased with increasing edge density of grassland patches (Pr(*β*_5_ > 0) = 0.973–0.982) until specific threshold points (5 km: *ϕ* = 19.8 m/ha, 95% CRI = 17.0–21.9 m/ha; 10 km: *ϕ* = 19.3 m/ha, 95% CRI = 17.0–21.9 m/ha; landscape with similar edge density depicted in Figure 4), after which abundance decreased with increasing edge density (Pr(*δ* < 0) = 0.989–0.993) (Table 3, Figure 5).

**TABLE 2:** Standardized results of the threshold models for percent grassland (GRASS) for each species (LEPC for lesser prairie chicken, GRPC for greater prairie chicken, RNEP for ring‐necked pheasant, and NOBO for northern bobwhite) and buffer size combination

Model combination		PDSI			PCP			TMAX			TMIN		
Species	Buffer (km)	Mean	Lower 95% CI	Upper 95% CI	Mean	Lower 95% CI	Upper 95% CI	Mean	Lower 95% CI	Upper 95% CI	Mean	Lower 95% CI	Upper 95% CI
LEPC	3	0.149	−0.019	0.311	−0.142	−0.295	0.015	−0.023	−0.167	0.122	−0.142	−0.288	0.002
LEPC	5	0.143	−0.028	0.320	−0.024	−0.187	0.142	0.014	−0.134	0.162	−0.150	−0.302	0.001
LEPC	10	0.149	−0.026	0.329	−0.058	−0.240	0.125	0.014	−0.134	0.171	−0.179	−0.332	−0.022
GRPC	3	0.110	−0.047	0.276	−0.174	−0.332	−0.019	−0.073	−0.198	0.049	−0.192	−0.362	−0.018
GRPC	5	0.101	−0.056	0.260	−0.154	−0.313	0.003	−0.089	−0.211	0.035	−0.194	−0.370	−0.022
GRPC	10	0.104	−0.050	0.260	−0.128	−0.288	0.033	−0.070	−0.194	0.057	−0.158	−0.335	0.015
NOBO	3	0.081	0.027	0.136	−0.006	−0.058	0.047	−0.130	−0.187	−0.072	0.210	0.158	0.263
NOBO	5	0.079	0.023	0.133	−0.003	−0.056	0.048	−0.134	−0.192	−0.077	0.211	0.158	0.262
NOBO	10	0.079	0.026	0.136	−0.006	−0.059	0.045	−0.131	−0.188	−0.074	0.211	0.158	0.263
RNEP	3	0.172	0.107	0.237	−0.271	−0.331	−0.208	0.035	−0.028	0.103	−0.140	−0.200	−0.078
RNEP	5	0.171	0.108	0.235	−0.265	−0.326	−0.202	0.036	−0.029	0.102	−0.122	−0.182	−0.061
RNEP	10	0.176	0.111	0.241	−0.283	−0.345	−0.221	0.051	−0.015	0.116	−0.102	−0.163	−0.040

Model combination		GRASS			δ			*ϕ*		
Species	Buffer (km)	Mean	Lower 95% CI	Upper 95% CI	Mean	Lower 95% CI	Upper 95% CI	Mean	Lower 95% CI	Upper 95% CI
LEPC	3	1.241	0.873	1.584	−2.042	−2.592	−1.523	0.265	0.026	0.519
LEPC	5	1.011	0.538	1.485	−1.844	−2.497	−1.175	0.217	−0.143	0.535
LEPC	10	0.129	−0.117	0.399	−2.732	−4.699	−0.603	1.418	0.742	2.018
GRPC	3	−0.422	−0.907	0.058	0.912	0.383	1.449	−0.345	−1.179	0.689
GRPC	5	−0.294	−0.617	0.022	0.762	0.314	1.221	−0.148	−0.703	0.508
GRPC	10	0.055	−0.389	0.300	−5.608	−10.577	1.314	1.183	−0.010	1.552
NOBO	3	−0.298	−2.777	0.548	−0.035	−3.808	3.496	−0.318	−2.239	1.754
NOBO	5	0.131	0.025	0.350	−2.223	−5.828	0.099	1.127	−1.224	1.841
NOBO	10	0.300	−0.051	1.864	−1.948	−5.422	0.305	0.925	−2.090	2.065
RNEP	3	0.009	−0.094	0.114	−1.127	−1.374	−0.870	0.471	0.347	0.625
RNEP	5	−0.001	−0.110	0.113	−1.135	−1.392	−0.873	0.422	0.261	0.594
RNEP	10	−0.152	−0.233	−0.076	−1.565	−1.934	−1.177	1.107	0.976	1.218

Note

Shown are mean effects of each variable and 95% credible intervals of effects of each variable. Weather variables included the Palmer Drought Severity Index (PDSI) of summer months with a 1‐year lag effect, precipitation index (PCP) of winter months in the winter prior to survey season, maximum temperature (TMAX) of summer months with a 1‐year lag effect, and minimum temperature (TMIN) of winter months in the winter prior to survey season. The intensification coefficient for the percent grassland threshold effect is designated δ, and the threshold point is *ϕ*.

**TABLE 3:** Standardized results of the threshold models for edge density (ED) for each species (LEPC for lesser prairie chicken, GRPC for greater prairie chicken, RNEP for ring‐necked pheasant, and NOBO for northern bobwhite) and buffer size combination

Model combination		PDSI			PCP			TMAX			TMIN		
Species	Buffer (km)	Mean	Lower 95% CI	Upper 95% CI	Mean	Lower 95% CI	Upper 95% CI	Mean	Lower 95% CI	Upper 95% CI	Mean	Lower 95% CI	Upper 95% CI
LEPC	3	0.098	0.098	0.280	−0.179	−0.180	−0.010	−0.025	−0.025	0.129	−0.202	−0.201	−0.053
LEPC	5	0.103	−0.078	0.281	−0.149	−0.317	0.024	−0.014	−0.168	0.137	−0.194	−0.343	−0.039
LEPC	10	0.100	−0.082	0.273	−0.133	−0.303	0.036	−0.012	−0.164	0.138	−0.191	−0.338	−0.037
GRPC	3	0.097	−0.054	0.249	−0.157	−0.305	−0.013	−0.097	−0.215	0.020	−0.198	−0.365	−0.030
GRPC	5	0.097	−0.057	0.096	−0.103	−0.254	−0.104	−0.104	−0.222	−0.103	−0.162	−0.336	−0.161
GRPC	10	0.082	−0.062	0.229	−0.109	−0.243	0.027	−0.112	−0.229	−0.001	−0.160	−0.326	−0.006
NOBO	3	0.083	0.028	0.136	−0.030	−0.083	0.021	−0.113	−0.171	−0.057	0.218	0.168	0.271
NOBO	5	0.081	0.027	0.135	−0.031	−0.083	0.024	−0.114	−0.173	−0.058	0.213	0.162	0.265
NOBO	10	0.081	0.026	0.135	−0.033	−0.088	0.022	−0.111	−0.172	−0.054	0.215	0.162	0.265
RNEP	3	0.145	0.084	0.208	−0.181	−0.243	−0.123	−0.045	−0.109	0.019	−0.161	−0.218	−0.103
RNEP	5	0.150	0.088	0.211	−0.184	−0.244	−0.123	−0.044	−0.108	0.019	−0.174	−0.231	−0.116
RNEP	10	0.161	0.098	0.222	−0.192	−0.254	−0.131	−0.027	−0.091	0.036	−0.178	−0.236	−0.119

Model combination		ED			δ			*ϕ*		
Species	Buffer (km)	Mean	Lower 95% CI	Upper 95% CI	Mean	Lower 95% CI	Upper 95% CI	Mean	Lower 95% CI	Upper 95% CI
LEPC	3	0.737	0.082	3.940	−1.899	−1.274	1.173	0.158	−0.465	2.196
LEPC	5	4.002	−0.281	6.941	−4.377	−7.728	−0.687	−1.007	−1.344	−0.688
LEPC	10	4.455	0.380	7.723	−4.696	−7.855	−0.696	−1.218	−1.500	−0.898
GRPC	3	−0.498	−0.692	−0.210	2.008	−0.574	4.955	1.365	−0.829	1.959
GRPC	5	−0.397	−2.425	−0.295	−0.010	−2.552	−0.245	0.345	−1.521	0.499
GRPC	10	2.730	1.456	4.075	−3.194	−4.602	−1.933	−1.204	−1.319	−1.083
NOBO	3	0.140	0.088	0.196	−7.457	−9.929	−4.825	1.758	1.707	1.811
NOBO	5	0.142	0.083	0.200	−2.954	−5.484	−0.456	1.681	1.476	1.932
NOBO	10	0.125	0.066	0.183	−2.404	−5.073	−0.406	1.709	1.357	1.964
RNEP	3	−0.028	−0.129	0.074	−1.084	−1.293	−0.877	0.359	0.230	0.562
RNEP	5	−0.033	−0.137	0.070	−1.015	−1.216	−0.816	0.280	0.163	0.462
RNEP	10	0.138	−0.050	0.328	−0.921	−1.147	−0.710	−0.222	−0.456	0.040

Note

Shown are mean effects of each variable and 95% credible intervals of effects of each variable. Weather variables included the Palmer Drought Severity Index (PDSI) of summer months with a 1‐year lag effect, precipitation index (PCP) of winter months in the winter prior to survey season, maximum temperature (TMAX) of summer months with a 1‐year lag effect, and minimum temperature (TMIN) of winter months in the winter prior to survey season. The intensification coefficient for the edge density threshold effect is designated δ, and the threshold point is *ϕ*.

Greater prairie chicken abundance was highest in years following high summer PDSI (i.e., wetter summers; Pr(*β*_1_ > 0) = 0.867–0.910), low summer TMAX (i.e., cooler summers; Pr(*β*_3_ < 0) = 0.869–0.974), low winter PCP (i.e., drier winters; Pr(*β*_2_ < 0) = 0.908–0.985), and low winter TMIN (i.e., cooler winters; Pr(*β*_4_ < 0) = 0.965–0.990) (Tables 2 and 3). At the 3‐ and 5‐km spatial scales, greater prairie chicken abundance initially decreased with increasing grassland (Pr(*β*_5_ < 0) = 0.978–0.982) until specific threshold points (3 km: *ϕ* = 65.3%, 95% CRI = 53.6%–79.8%; 5 km: *ϕ* = 64.8%, 95% CRI = 56.7%–74.4% grassland), after which abundance increased with increasing grassland (Pr(*δ* > 0) = 0.997–1) (Table 2, Figure 3). At the 10‐km spatial scale, greater prairie chicken abundance initially increased with increasing grassland (Pr(*β*_5_ > 0) = 0.740) until the threshold point (*ϕ* = 82.1%, 95% CRI = 63.9%–87.7%), after which abundance decreased with increasing grassland (Pr(*δ* < 0) = 0.753) (Table 2, Figure 3). However, few data points with a percent grassland greater than this threshold existed. At the 3‐km spatial scale, greater prairie chicken abundance initially decreased with increasing edge density of grassland patches Pr(*β*_5_ < 0) = 0.989) until the threshold point (*ϕ* = 67.5 m/ha, 95% CRI = 40.4–74.8 m/ha), after which abundance increased with increasing edge density (Pr(*δ* > 0) = 0.932) (Table 3, Figure 5). However, few data points with an edge density greater than this threshold point existed. At the 5‐km spatial scale, greater prairie chicken abundance decreased with increasing edge density of grassland patches (Pr(*β*_5_ < 0) = 0.913). At the 10‐km spatial scale, greater prairie chicken abundance initially increased with increasing edge density of grassland patches (Pr(*β*_5_ > 0) = 1) until the threshold point (*ϕ* = 38.6 m/ha, 95% CRI = 37.2–40.1 m/ha; landscape with similar edge density depicted in Figure 4), after which abundance decreased with increasing edge density (Pr(*δ* < 0) = 1) (Table 3, Figure 5).

Northern bobwhite abundance was highest in years following high summer PDSI (i.e., wetter summers; Pr(*β*_1_ > 0) = 0.998–0.999), low summer TMAX (i.e., cooler summers; Pr(*β*_3_ < 0) = 1), low winter PCP (i.e., drier winters; Pr(*β*_2_ < 0) = 0.548–0.883), and high winter TMIN (i.e., warmer winters; Pr(*β*_4_ > 0) = 1) (Tables 2 and 3). At the 3‐km spatial scale, northern bobwhite abundance increased with increasing grassland (Pr(*β*_5_ > 0) = 0.728) (Table 2, Figure 3). At the 5‐ and 10‐km spatial scales, northern bobwhite abundance initially increased with increasing grassland (Pr(*β*_5_ > 0) = 0.984–0.996) until specific threshold points (5 km: *ϕ* = 77.0%, 95% CRI = 33.3%–90.3%; 10 km: *ϕ* = 69.7%, 95% CRI = 18.1%–89.2% grassland), after which abundance decreased with increasing grassland (Pr(*δ* < 0) = 0.961–0.983) (Table 2, Figure 3). However, few data points with an edge density greater than these threshold points existed. At the 3‐, 5‐, and 10‐km spatial scales, northern bobwhite abundance initially increased with increasing edge density of grassland patches (Pr(*β*_5_ > 0) = 1) until specific threshold points (3 km: *ϕ* = 79.5 m/ha, 95% CRI = 78.7–80.2 m/ha; 5 km: *ϕ* = 78.6 m/ha, 95% CRI = 75.6–82.4 m/ha; 10 km: *ϕ* = 77.5 m/ha, 95% CRI = 72.5–81.1 m/ha; landscape with similar edge density depicted in Figure 4), after which abundance decreased with increasing edge density (Pr(*δ* < 0) = 0.998–1) (Table 3, Figure 5).

Ring‐necked pheasant abundance was highest in years following high summer PDSI (i.e., wetter summers; Pr(*β*_1_ > 0) = 1), low winter PCP (i.e., drier winters; Pr(*β*_2_ < 0) = 1), and low winter TMIN (i.e., cooler winters; Pr(*β*_4_ < 0) = 0.999–1) (Tables 2 and 3). The effect of summer TMAX on ring‐necked pheasant abundance varied among models (Pr(*β*_3_ < 0) = 0.065–0.918) (Tables 2 and 3). At the 3‐ and 5‐km spatial scales, ring‐necked pheasant abundance did not initially differ with increasing grassland (Pr(*β*_5_ > 0) = 0.485–0.564) until specific threshold points (3 km: *ϕ* = 48.6%, 95% CRI = 46.4%–51.3%; 5 km: *ϕ* = 48.2%, 95% CRI = 45.5%–51.0% grassland), after which abundance decreased with increasing grassland (Pr(*δ* < 0) = 1) (Table 2, Figure 3). At the 10‐km spatial scale, ring‐necked pheasant abundance initially decreased with increasing grassland (Pr(*β*_5_ < 0) = 1) until a threshold point (*ϕ* = 59.4%, 95% CRI = 57.4%–61.2% grassland), after which abundance decreased more severely with increasing grassland (Pr(*δ* < 0) = 1) (Table 2, Figure 3). At the 3‐ and 5‐km spatial scales, ring‐necked pheasant abundance initially decreased with increasing edge density of grassland patches (Pr(*β*_5_ < 0) = 0.706–0.731) until specific threshold points (3 km: *ϕ* = 52.1 m/ha, 95% CRI = 50.1–55.2 m/ha; 5 km: *ϕ* = 51.1 m/ha, 95% CRI = 49.3–53.9 m/ha; Figure 4), after which abundance decreased more severely with increasing edge density (Pr(*δ* < 0) = 1) (Table 3, Figure 5). At the 10‐km spatial scale, ring‐necked pheasant abundance increased with increasing edge density of grassland patches (Pr(*β*_5_ > 0) = 0.925) until the threshold point (10 km: *ϕ* = 43.0 m/ha, 95% CRI = 39.5–46.8 m/ha; landscape with similar edge density depicted in Figure 4), after which abundance decreased with increasing edge density (Pr(*δ* < 0) = 1) (Table 3, Figure 5).

## 4 DISCUSSION

Abundance estimates from point counts are an integral part of avian monitoring efforts that allow researchers to quantify population trends and assess the effects of environmental covariates on abundance (Sauer et al., 2013). We found evidence of an overall decline in abundance of lesser and greater prairie chickens between the 1978–2014 and 1996–2014 survey periods, respectively, although there was interannual variation in population trends over this period. This result supports previous assessments of prairie chicken declines in the state (Jensen et al., 2000; Nasman et al., 2018; Pitman, 2014) and throughout the Great Plains (Garton et al., 2016; Johnsgard, 2002; McNew et al., 2011). We did not find evidence of an overall decline in abundance of northern bobwhites or ring‐necked pheasants between the 1997–2015 and 1998–2015 survey periods, respectively, although there was large interannual variability. While both northern bobwhites and ring‐necked pheasants have experienced contemporary declines in Kansas and throughout much of the species’ respective ranges (Hernández et al., 2013; Sauer et al., 2013), populations of these species in Kansas were relatively stable during our survey years (1997–2015 for ring‐necked pheasants and 1998–2015 for northern bobwhites; Prendergast, 2018a, 2018b).

It is important to understand species‐specific responses in abundance related to weather events when projecting how species may respond to future projected climate change. In the Great Plains of the United States, climate change is expected to increase intensity and frequency of drought, resulting in significantly drier conditions in the latter half of the 21st century (Cook et al., 2015). We found that all four focal species had greater abundances following wetter summers, and greater prairie chickens and northern bobwhites had greater abundances following cooler summers. Other studies have found extreme summer temperatures and drought to negatively affect prairie chicken, northern bobwhite, and ring‐necked pheasant reproductive success (Carroll et al., 2015, 2017; Fritts et al., 2018; Laskowski et al., 2017; Ross et al., 2016a, 2016b). These species may therefore be particularly at risk to future changes in climate.

The variation in the effects of percent grassland on abundance of our four focal species is likely attributed to different life‐history strategies among species. For example, lesser prairie chickens generally occupy habitats containing mid‐ and tall grasses throughout the year (Haukos & Zavaleta, 2016; Jones, 1963) and conversion of grassland to cropland is often attributed to declines in abundance of both lesser and greater prairie chickens (Hagen et al., 2004; Johnson et al., 2020). However, there is evidence that lesser prairie chickens use croplands, particularly during winter months, and presence of some cropland in the landscape can increase abundance of this species, likely through providing winter forage (Hagen et al., 2007; Ross et al., 2016b). Lesser prairie chickens also benefit from landscape heterogeneity, so the presence of cropland may additionally help create habitat mosaics that support the different landscape types required throughout different life stages (Robinson et al., 2019). While greater prairie chickens avoid cropland during the breeding season (Raynor et al., 2019), similarities in life history and habitat selection to lesser prairie chickens likely result in greater prairie chickens receiving similar benefits from access to cropland during winter months. Northern bobwhites and ring‐necked pheasants often occupy habitat in or near agricultural land (Hagen et al., 2007; Janke et al., 2015). Responses of ring‐necked pheasants to increasing grassland in particular highlight the importance of cropland in conjunction with grassland as a habitat source for this species. Prior research has demonstrated ring‐necked pheasants commonly use cropland and grassland adjacent to cropland throughout the year, further suggesting both may be important to optimizing ring‐necked pheasant abundance in a landscape, though crop type may be an important consideration (Basore et al., 1986; Clark et al., 1999; Coates et al., 2016; Hagen et al., 2007).

In addition to habitat quantity, configuration of habitat patches is also an important driver in grassland bird abundance (Fuhlendorf et al., 2002; Hernández et al., 2013). Greatest abundance of our focal species occurred not only when the landscape contained an optimal amount of grassland, but also when grassland patches were configured in shapes with the optimal (i.e., threshold point) amount of edge at the respective spatial scales. The presence of some edge habitat (small amounts of edge for lesser prairie chickens, intermediate amounts of edge for greater prairie chickens and ring‐necked pheasants, and large amounts of edge for northern bobwhites) allows for interactions of each species with cropland, which likely provides additional winter forage for all four species. Lesser prairie chickens sometimes use cultivated fields adjacent to grasslands as lekking sites, and grassland edge may provide these habitats as well (Hagen et al., 2004; Jamison et al., 2002). Northern bobwhites and ring‐necked pheasants are generally categorized as more edge‐dwelling than prairie chickens, often selecting habitat in close association with cropland (Hagen et al., 2007; Janke et al., 2015). In particular, edge habitat between grassland and cropland patches likely increases the availability of shrubby escape cover along field edges, which has been shown to increase survival during winter months for northern bobwhites and ring‐necked pheasants (Gabbert et al., 1999; Janke et al., 2015).

Habitat characteristics at both fine‐ and landscape‐level scales are important drivers of grassland bird abundance (Doherty et al., 2010; Fuhlendorf et al., 2002; Williams et al., 2004), yet studies often focus on one scale, potentially providing an incomplete understanding of habitat needs of species of interest for managers (Doherty et al., 2010; Kristan & Scott, 2006). The effects of percent grassland and edge density for both lesser and greater prairie chickens differed by scale. In both species, managing for optimal grassland cover is likely most important at the fine and intermediate scales, as the effect of percent grassland on abundance was strongest at these scales. Managing for edge habitat is likely most important at the intermediate‐ and landscape‐level scales for lesser prairie chickens and landscape‐level scale for greater prairie chickens, as threshold effects were only evident at these scales. Without considering multiple spatial scales, we would have an incomplete understanding of how these landscape characteristics affected prairie chicken abundance. The effects of percent grassland and edge density on northern bobwhite and ring‐necked pheasant abundance were similar across spatial scales. However, northern bobwhite and ring‐necked pheasant home ranges are generally smaller than prairie chicken home ranges (Applegate et al., 2002; Haukos & Zavaleta, 2016; Janke & Gates, 2012; Patten et al., 2011), so changes in response may occur at finer scales than our smallest buffer size.

While all species likely benefit from a mosaic of grassland and cropland in the landscape, differences in responses to proportions and configurations of various land cover may limit conservation potential in an umbrella species management plan. Instead, managers could manage for grassland cover (e.g., near the percent grass threshold point for lesser prairie chickens and northern bobwhites, near or greater than the threshold points for greater prairie chickens, and near or less than the threshold points for ring‐necked pheasants) and configurations of grassland patches (e.g., near the edge density threshold points for all four species) that optimize abundance across all or a combination of species, but are not ideal for any one species.

## 5 CONCLUSION

Decreasing quantity and quality of grasslands in the Great Plains due to expansion and intensification of agriculture has resulted in dramatic declines in grassland bird abundance. Conservation of grassland birds is often focused around restoring grassland, through programs such as the Conservation Reserve Program. While all grassland birds likely require some minimum amount of grassland, many species have habitat requirements that vary throughout the year and at different life cycle stages, and thus benefit from heterogeneous landscapes. We found landscape heterogeneity to be important for lesser and greater prairie chickens, ring‐necked pheasants, and northern bobwhites, with abundance of each species optimized in landscapes that represented a grassland and cropland mosaic. When managing such landscapes, managers may face trade‐offs when habitat needs of multiple species conflict, or conservation priorities of species differ. For example, managers may choose to manage more heterogeneous landscapes for ring‐necked pheasants and northern bobwhite and landscapes with more intact grassland for lesser or greater prairie chickens, or choose to prioritize prairie chickens over northern bobwhites and ring‐necked pheasant due to differences in conservation concern. We demonstrated a novel approach for estimating landscape conditions needed to optimize abundance across multiple species at a variety of spatial scales, thus improving conservation potential across a landscape. This framework will help inform conservation managers, so they may simultaneously develop conservation plans for multiple species of interest.

## CONFLICT OF INTERESTS

We have no competing interests.

## AUTHOR CONTRIBUTION

**Alexander R. Schindler:** Conceptualization (equal); Data curation (lead); Formal analysis (lead); Investigation (equal); Visualization (lead); Writing‐original draft (lead); Writing‐review & editing (lead). **David A. Haukos:** Conceptualization (equal); Investigation (equal); Writing‐review & editing (supporting). **Christian A. Hagen:** Conceptualization (equal); Investigation (equal); Writing‐review & editing (supporting). **Beth E. Ross:** Conceptualization (equal); Formal analysis (supporting); Investigation (equal); Project administration (lead); Supervision (lead); Writing‐review & editing (lead).

## Supporting information

Figures S1‐S2

Data S1

## Data Availability

Data are available on Dryad (https://doi.org/10.5061/dryad.c59zw3r5w).
