# Supplementary material for: A multispecies approach to manage effects of land cover and weather on upland game birds
Source: Ecol Evol. 2020 Nov 19;10(24):14330–45. doi: 10.1002/ece3.7034 (PMC7771187; doi:10.1002/ece3.7034)
Supplement: Supplementary file 1 — Figures S1‐S2 [file ECE3-10-14330-s001.docx]

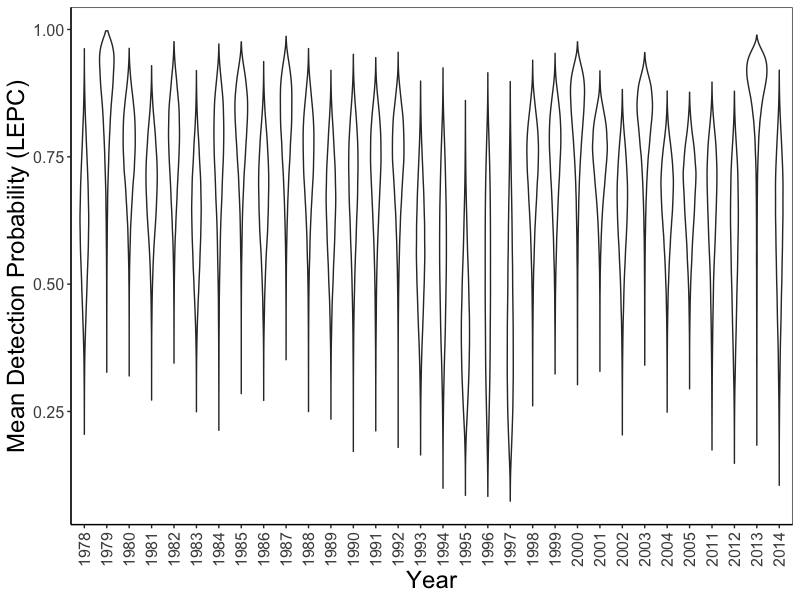


Figure S1. Mean detection probabilities of lesser prairie-chickens (LEPC) on leks across all routes for each year included in our models.


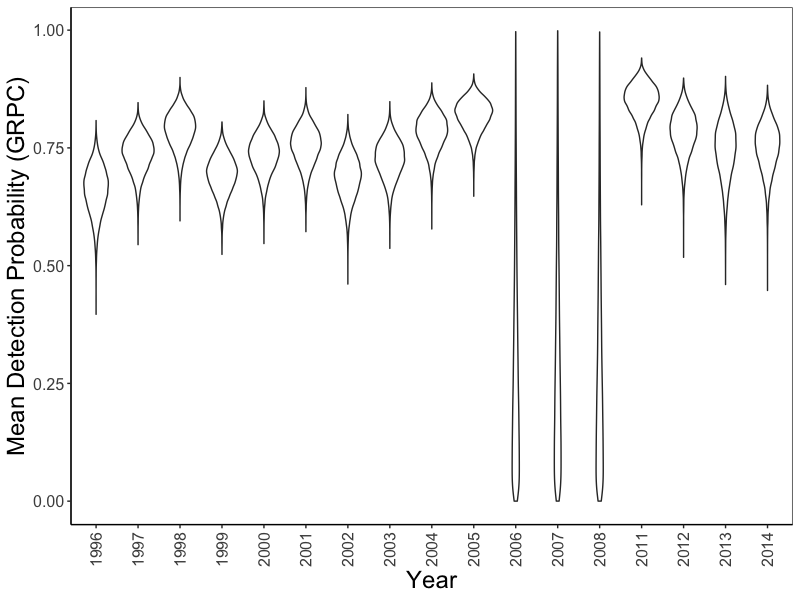


Figure S2. Mean detection probabilities of greater prairie-chickens (GRPC) on leks across all routes for each year included in our models.
